# Rationale for an Association Between PD1 Checkpoint Inhibition and Therapeutic Vaccination Against HIV

**DOI:** 10.3389/fimmu.2018.02447

**Published:** 2018-10-23

**Authors:** Gilberto Filaci, Daniela Fenoglio, Lucia Taramasso, Francesco Indiveri, Antonio Di Biagio

**Affiliations:** ^1^Centre of Excellence for Biomedical Research and Department of Internal Medicine, University of Genoa, Genoa, Italy; ^2^Biotherapy Unit, Ospedale Policlinico San Martino, Genoa, Italy; ^3^Infectious Disease Unit, Ospedale Policlinico San Martino, Genoa, Italy

**Keywords:** HIV, Treg, immune checkpoints, HIV vaccine, PD1

## Abstract

The pathogenesis of HIV immunodeficiency is mainly dependent on the cytopatic effects exerted by the virus against infected CD4+ T cells. However, CD4+ T cell loss cannot be the only pathogenic factor since severe opportunistic infections may develop in HIV infected patients with normal CD4+ T cell counts and since the recent START study indicated that absolute CD4+ T cell counts are not predictive for AIDS and non-AIDS events. Recently our group demonstrated that CD8+CD28-CD127lowCD39+ regulatory T lymphocytes, previously found highly concentrated within tumor microenvironment, circulate with elevated frequency in the peripheral blood of HIV infected patients. Here, we show that these cells, that at least in part are HIV specific, express the PD1 immune checkpoint. Based on these evidences and considerations, in this Perspective article we speculate on the opportunity to treat HIV infected patients with anti-PD1 immune checkpoint inhibitors as a way to counteract the T regulatory cell compartment and to unleash virus-specific immune responses. In order to potentiate the immune responses against HIV we also propose the potential utility to associate immune checkpoint inhibition with HIV-specific therapeutic vaccination, reminiscent of what currently applied in oncologic protocols. We suggest that such an innovative strategy could permit drug-sparing regimens and, perhaps, lead to eradication of the infection in some patients.

The pathogenesis of HIV immunodeficiency is dependent on the cytopatic effects exerted by the virus against infected CD4+ T cells and to subsequent CD4+ T cell loss (1, 2). Some aspects of the disease remain unexplained as the case of HIV infected patients with normal CD4+ T cell counts after anti-retroviral therapy (ART) initiation who develop severe opportunistic infections (3, 4), or that of HIV infected patients with reduced CD4+ T cell counts who do not show immunodeficiency manifestations (5). Accordingly, the results of the recent START study indicated that absolute CD4+ T cell counts are not predictive for AIDS and non-AIDS events since these events may occur in ART treated patients with absolute CD4 counts >500 cells/μl (6). Searching for other pathogenic mechanisms, we focused our attention on regulatory T lymphocytes (Treg). The role of these cells in HIV immunodeficiency pathogenesis is still unclear since studies on alterations of CD4+ Treg in HIV infected patients led to controversial results (7–17). Hence, we took into consideration a different subset of Treg constituted by CD8+ Treg (18). CD8+ Treg, in particular those expressing the CD8+CD28-CD127lowCD39+ phenotype, are regulatory T lymphocytes found highly concentrated within tumor microenvironment, where they can exert remarkable immunosuppressive activity due to their capacity to target T cell proliferation and cytotoxicity (19–21). We investigated on the presence of these cells in the circulation of HIV-infected patients, and on possible correlations between their frequency and markers of disease activity. The results of this study demonstrated that HIV-infected patients have elevated circulating levels of functional CD8+CD28-CD127lowCD39+ Treg, the majority of which is antigen-specific for HIV proteins. This observation is remarkable since these cells are virtually absent from the circulation of healthy subjects (19, 21). In HIV patients, their frequency post-ART correlates with HIV-RNA, CD4+ T cell count, and immune activation markers, suggesting their pathogenic involvement in AIDS or non-AIDS related complications. Moreover, their increase after initiation of ART heralds a lack of virological or clinical response (i.e., appearance of co-morbidity): hence their monitoring is clinically relevant (22).

Further studies from our group show that CD8+CD28-CD127lowCD39+ Treg stably and consistently express PD1 (Figure 1 and Supplemental Material). PD1 is a member of immune checkpoints (23–26). These are molecules expressed by immune cells in order to control the immune responses. In particular, PD1 is mainly expressed by T lymphocytes at advanced stage of maturation, i.e., effector memory and terminally-differentiated effector memory cells. Hence, it is involved in the control of the effector phase of the immune response. Indeed, PD1+ T cells are present within tumor infiltrating lymphocytes, suggesting that PD1 expression contributes to tumor immune evasion (24, 27). In HIV-infected patients CD4+PD1+ T cells constitute the major HIV cell reservoir (28, 29). Interestingly, PD1+ T cell frequency is elevated in HIV-infected patients and reduces after beginning of the treatment, a behavior reminiscent of what occurs to circulating CD8+CD28-CD127loCD39+ Treg frequency (30, 31). In HIV-infected patients, the presence of increased frequency of PD1+ T cells, including abnormally expanded CD8+CD28-CD127loCD39+PD1+ Treg, could be involved in generating immunodeficiency and hampering anti-virus immune responses. Hence, the fact that expansion of CD8+ Treg and of PD1+ T lymphocytes co-exist in both tumors and HIV infection envisages a pathogenic crossing between the two pathologic conditions. This suggests that targeting PD1+ T cells through a specific checkpoint inhibitor could be a useful therapeutic strategy for HIV infection borrowed from anti-cancer protocols, based that recent trials support the safety of this approach (32, 33). The potential efficacy of this strategy is further supported by previous studies in which a PD1 inhibitor was administered to non-human primates. In these studies, anti-PD1 treatment of uninfected animals co-immunized with a SIV-gag adenovirus vector vaccine enhanced the frequency of gag-specific T cells (34), while treatment of SIV infected macaques increased SIV-specific immune response, decreased viral load and prolonged survival (35). Accordingly, administration of anti-PD-L1 monoclonal antibodies to ART-treated SIV infected macaques allowed the maintenance of a lower viral load after ART suspension than in non-administered animals (36).

**Figure 1:**
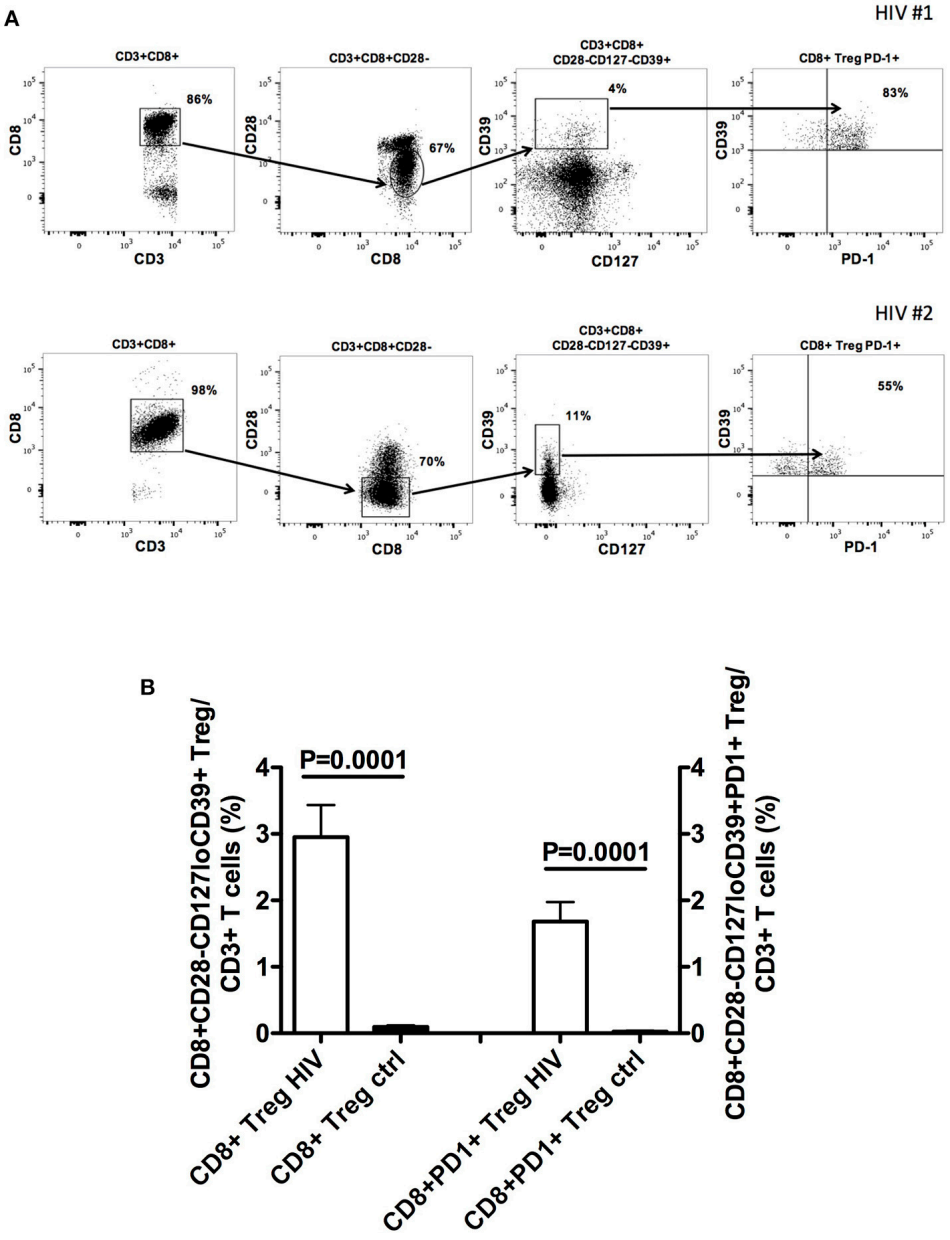
CD8+CD28−CD127loCD39+PD1+ Treg frequency in the peripheral blood of naïve HIV-infected patients. **(A)** The gating strategy for the analysis of CD8+CD28−CD127loCD39+PD1+ Treg frequency in the circulation of two representative naïve HIV-infected patients is shown. **(B)** Comparison between the mean frequency of CD8+CD28−CD127loCD39+ Treg as well as that of CD8+CD28−CD127loCD39+PD1+ Treg in the circulation of 22 naïve HIV-infected patients and those of 22 healthy controls. Detailed information on patient population and methods is provided in the Supplementary File—Patients and Methods.

However, in the majority of cancer patients the activity of the sole checkpoint inhibitor is not sufficient to provide a robust therapeutic effect. Hence, association of checkpoint inhibitors with therapeutic vaccination is currently proposed as optimal way for inducing/reinforcing anti-tumor immune responses in the absence of abnormal and detrimental regulatory mechanisms (37, 38). Interestingly, the development of a vaccine against HIV has been recently evoked as urgent medical need, notwithstanding the efficacy of ART. In fact, HIV pandemia is so wide (more than 36 million infected people, about 1.8 million people newly infected each year) that the costs for life-long ART are huge for government health care systems. Hence, therapeutic vaccination has been proposed as a preferential therapeutic tool for corroborating ART activity possibly through the eradication of HIV-1 latent reservoirs (39, 40). Indeed, an optimal vaccine against HIV should elicit both virus-specific cytotoxic CD8+ T cells and neutralizing antibodies in order to kill virus-infected cells, that constitute the viral reservoir, and to avoid spreading of infection by viral particles released by already infected cells. Among HIV antigens, gag has been considered a useful immunogen for vaccine preparation since the presence of elevated titers of antibodies against gag, but not against other HIV antigens, correlated with reduced viremia in HIV infected patients (41). Disappointingly, all vaccination trials so far performed in prophylactic or therapeutic settings, including those using gag as immunogen, did not achieve brilliant clinical results (42). Now, the finding of an abnormal expansion of CD8+CD28-CD127loCD39+PD1+ Treg in these patients suggests that such defective activity of HIV vaccines could have a bi-faceted origin, related to inner deficiency of vaccine immunogenicity and/or to the generalized immunosuppressive effect exerted by CD8+ Treg. Accordingly, a DNA vaccine based on a chimeric gene product fusing PD1 and gag moieties induced high frequency of gag-specific cytotoxic CD8+ T cells associated with high titers of virus-specific antibodies, conferring remarkable protection against mucosal challenge with vaccinia gag viruses in experimental animals (43).

These considerations may constitute a robust rationale for a combination therapy associating anti-PD1 checkpoint inhibition and therapeutic vaccination in ART treated HIV-infected patients. Our hypothesis is that early co-administration of ART, checkpoint inhibition, and vaccination in recently HIV-infected patients could allow to take advantage of the synergic effect of the three-faceted approach when the HIV latent reservoir is not yet consolidated. We expect that such an innovative strategy could lead to the onset of HIV specific immune responses more effective than those spontaneously developed in the absence of Treg inhibition, since unleashed by the regulatory control of PD1+ Treg. Hopefully, this strategy could permit drug-sparing regimens and, perhaps, lead to eradicate the infection in some patients.

## Ethics statement

The study was carried out in compliance with the Helsinki Declaration and was approved by the Ethics Committee of the San Martino Hospital in Genoa, Italy (P.R.251REG2014). All enrolled patients provided written informed consent.

## Author contributions

GF, FI, and AD wrote the manuscript; DF performed the phenotypic analyses; LT enrolled and clinically managed the patients.

### Conflict of interest statement

The authors declare that the research was conducted in the absence of any commercial or financial relationships that could be construed as a potential conflict of interest.
