# Supplementary material for: Rationale for an Association Between PD1 Checkpoint Inhibition and Therapeutic Vaccination Against HIV
Source: Front Immunol. 2018 Oct 23;9:2447. doi: 10.3389/fimmu.2018.02447 (PMC6232923; doi:10.3389/fimmu.2018.02447)
Supplement: Supplementary file 1 [file Table_1.docx]

| **Supplementary Table 1. Clinical data of HIV infected patients enrolled in the study.** | | | | |
| --- | --- | --- | --- | --- |
| **Patient #** | **Sex** | **Age (years)** | **Absolute CD4+ T cell N./μl** | **Viral load**  **(copies/ml)** |
| 1 | F | 42 | 287 | 103400 |
| 2 | M | 51 | 43 | 2649000 |
| 3 | F | 46 | 100 | 84200 |
| 4 | M | 24 | 632 | 28100 |
| 5 | M | 48 | 167 | 45400 |
| 6 | M | 24 | 145 | 770000 |
| 7 | M | 69 | 351 | 24000 |
| 8 | M | 55 | 198 | 118100 |
| 9 | M | 39 | 22 | 285000 |
| 10 | M | 42 | 933 | 1300 |
| 11 | M | 45 | 662 | 33900 |
| 12 | F | 59 | 173 | 8900 |
| 13 | M | 54 | 33 | 50981 |
| 14 | M | 36 | 72 | 18000000 |
| 15 | M | 53 | 436 | 17900 |
| 16 | F | 31 | 197 | 86000 |
| 17 | F | 36 | 251 | 60000 |
| 18 | M | 78 | 369 | 262500 |
| 19 | F | 32 | 359 | 2627000 |
| 20 | M | 42 | 212 | 3400 |
| 21 | M | 29 | 501 | 14100 |
| 22 | M | 26 | 228 | 105400 |
