# Supplementary material for: Rationale for an Association Between PD1 Checkpoint Inhibition and Therapeutic Vaccination Against HIV
Source: Front Immunol. 2018 Oct 23;9:2447. doi: 10.3389/fimmu.2018.02447 (PMC6232923; doi:10.3389/fimmu.2018.02447)
Supplement: Supplementary file 2 [file Data_Sheet_1.docx]

**Supplementary Material – Patients and Methods**

**Patients**

This was an observational, longitudinal, multicenter study. Twenty-two naïve patients with new diagnosis of HIV infection were enrolled in 8 Infectious Diseases Units located in North-Western Italy (Liguria and Piedmont). Plasma samples from each patient were collected at baseline before treatment administration.

The study was carried out in compliance with Helsinki Declaration and approved by the Ethical Committee of San Martino Hospital in Genoa (P.R.251REG2014). All enrolled patients provided written informed consent.

**Monoclonal antibodies (mAb) and immunofluorescence analyses**

Immunofluorescence analyses were performed on fresh blood samples. One hundred μl of fresh blood were incubated with specific fluorochrome-conjugated monoclonal antibodies (mAbs) at 4 °C for 30 min in the dark.

The following mAbs were used to analyze CD8+CD28-CD127loCD39+ Treg cells: phycoerythrin (PE) anti-CD127, clone HIL-7R-M21 (BD Biosciences), Peridinin Chlorophyll Protein Complex-cyanin 5.5 (PerCP-Cy5.5)-conjugated anti-CD28, clone CD28.2 (Biolegend), allophycocianin (APC)-conjugated anti-CD39, clone TU66 (BD Biosciences), Brilliant Violet (BV) 421-conjugated anti-CD8, clone RPA-T8 (BD Biosciences), APC-H7-conjugated anti-CD4, clone RPA-T4 (BD Biosciences), BV510-conjugated anti-CD3, clone UCHT1 (BD Biosciences), BV786-conjugated anti-PD1, clone EH12.1 (BD Biosciences).

Then, 500 μl of Facs Lysing solution (BD Biosciences) were added and incubated for 5 min in order to lyse red cells. At the end of incubation, the cells were washed with 1 ml of PBS-BSA 0.01% and resuspended in 300 μl of PBS. The samples were analyzed by a BD Fortessa X20 flow cytometer (BD Biosciences) using the BD FACSDiva™ software version 8.0 (BD Biosciences).
